# The functional near infrared spectroscopy applications in children with developmental diseases: a review

**DOI:** 10.3389/fneur.2025.1495138

**Published:** 2025-06-17

**Authors:** Jing Wang, Zhuo Zou, Haoyu Huang, Jinting Wu, Xianzhao Wei, Shuyue Yin, Yingjuan Chen, Yun Liu

**Affiliations:** Department of Rehabilitation, Kunming Children's Hospital, Kunming Medical University, Kunming, Yunnan, China

**Keywords:** functional near infrared spectroscopy, children, developmental diseases, hemodynamics, research progress

## Abstract

This review provides a comprehensive synthesis of the application of functional near-infrared spectroscopy (fNIRS) in pediatric developmental disorders, with a particular emphasis on its potential for clinical translation. fNIRS is a portable and non-invasive brain imaging technique that detects the relative concentration changes of oxyhemoglobin (HbO_2_), deoxyhemoglobin (HbR), and total hemoglobin in the cerebral cortex. These measurements effectively reflect cortical activation, making fNIRS a valuable tool in the field of pediatric neurodevelopmental research. The inherent resistance of fNIRS to interference, coupled with its adaptability to naturalistic settings, renders it particularly well-suited for pediatric populations. In this context, we undertook a meticulous and comprehensive literature search, employing predefined strategies and stringent inclusion/exclusion criteria (which are elaborated upon in the text). Our aim was to identify and review fNIRS studies across a wide range of developmental disorders. These disorders encompass cerebral palsy (CP), autism spectrum disorder (ASD), attention deficit hyperactivity disorder (ADHD), conditions related to preterm infants, hypoxic–ischemic encephalopathy (HIE), and idiopathic language disorders. Our synthesis uncovers distinct hemodynamic patterns associated with specific developmental disorders. For example, autism spectrum disorder (ASD) is marked by atypical activation within social brain networks, whereas attention deficit hyperactivity disorder (ADHD) is characterized by diminished activation in the prefrontal cortex. These findings not only shed light on the neurophysiological foundations of these disorders but also highlight the potential of fNIRS as a diagnostic biomarker. This review aims to inform the clinical application of fNIRS by providing a critical evaluation of its mechanistic insights and potential clinical pathways, thereby advancing its role in the diagnosis and management of developmental disorders.

## 1 Introduction

In the field of pediatric rehabilitation, many children experience varying degrees of abnormal brain development and dysfunction, which can significantly impair their learning and daily living. Early and effective rehabilitation interventions for these children can substantially enhance their functional abilities and overall quality of life. Therefore, the early detection of abnormal brain development and dysfunction in children, followed by timely rehabilitation treatment, is an urgent social issue that demands immediate attention and resolution (1). Nowadays, there are many ways to detect neurological diseases, such as functional magnetic resonance imaging (functional magnetic resonance imaging, fMRI), positron emission tomography (positron emission tomography, PET), electroencephalogram (electroencephalogram, EEG), and functional near infrared spectroscopy(fNIRS). Each of these tests has its own characteristics (Table 1) (2). fNIRS is an emerging non-invasive optical imaging technology that can indirectly reflect neural activity in the brain by detecting real-time levels of HbO_2_ and reduced HbR levels in the cerebral cortex (3). fNIRS is highly user-friendly and convenient to operate, with strong anti-interference capabilities and high compatibility. It imposes minimal requirements on the examination environment, allowing for assessments in a wide range of natural settings, such as schools or hospitals. Notably, it can even be conducted while infants and young children are held in their parents’ arms. These features make it particularly suitable for studying infants, young children, and those with developmental disorders. As such, fNIRS stands out as one of the most promising methods for investigating pediatric brain function (3). Currently, there is a growing body of research exploring the application of fNIRS in studying childhood developmental disorders. However, the development of fNIRS in this specific area remains inadequate. Against this backdrop, this paper reviews the current application and research status of fNIRS in the study of children’s developmental disorders. It elucidates the mechanisms underlying fNIRS in these disorders and aims to enhance the diagnostic and therapeutic approaches for pediatric developmental disorders. Ultimately, this review seeks to provide valuable insights and references for the clinical application and in-depth research of fNIRS in the context of children’s developmental disorders. At present, more and more studies on the application of fNIRS in childhood developmental disorders, but the development of fNIRS in childhood developmental disorders is lacking. Therefore, this paper reviews the application and research status of fNIRS in children’s developmental disorders, and explains the mechanism of fNIRS in children’s developmental disorders, improve the diagnosis and treatment level of children’s developmental disorders, in order to provide reference for the clinical application and in-depth research of fNIRS in children’s developmental disorders.

**Table 1:** Comparison of fNIRS with other neuroimaging methods (2).

Neuroimaging methods	Detection parameters	Spatial resolution	Temporal resolution	Anti motion interference	Anti-electromagnetic interference
fMRI	Hemodynamic response: Bold signal	3 mm	0–2 Hz	weak	weak
PET	glucose metabolism	4 mm	< 0.1 Hz	weak	weak
EEG	electrical nerve activity	6–9 cm	> 1,000 Hz	weak	weak
fNIRS	Hemodynamic response:HbO_2_/HbR	2–3 cm	0–10 Hz	strong	strong

## 2 Methods

**Figure 1:**
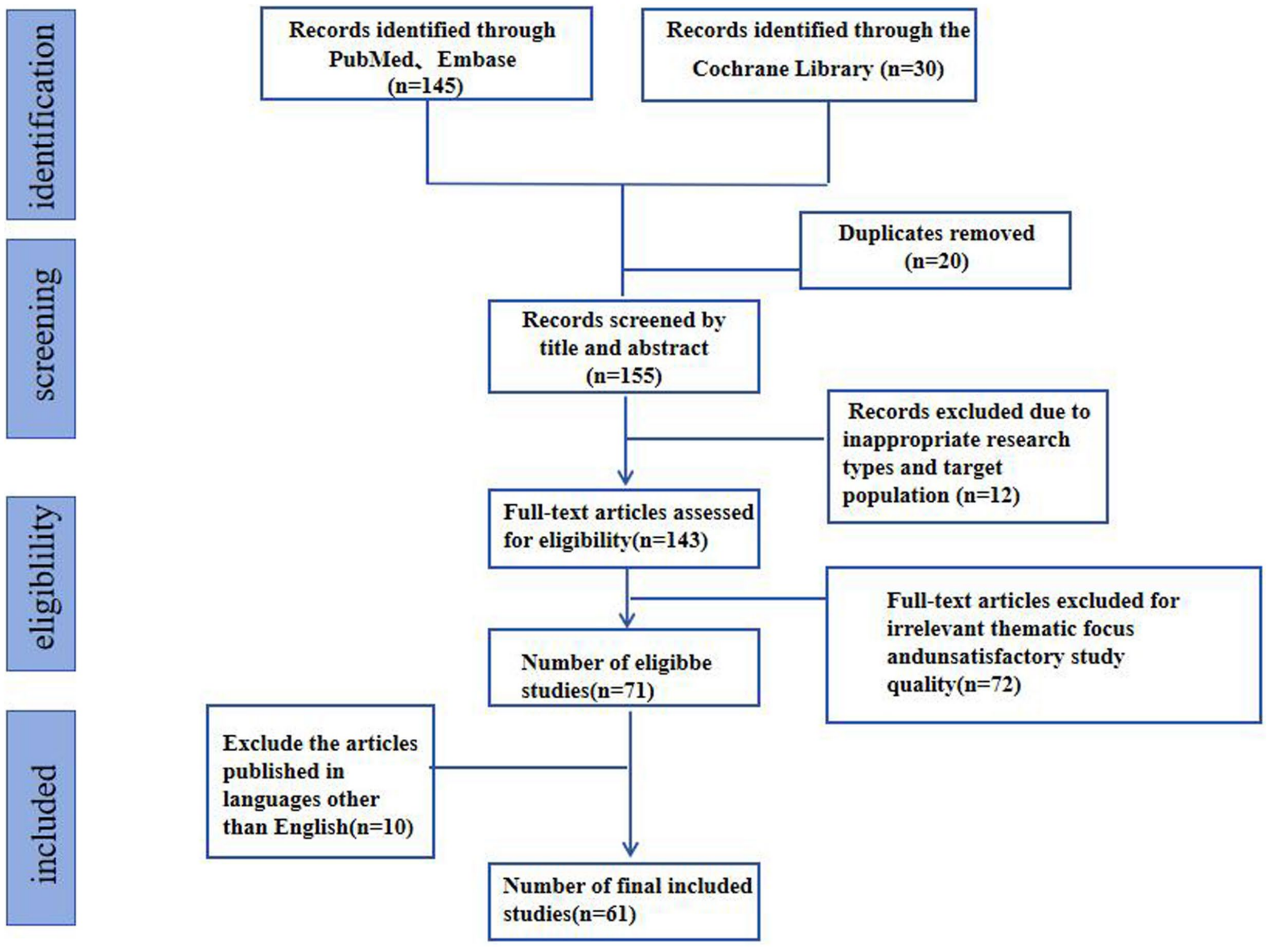
PRISMA (Preferred Reporting Items for Systematic Reviews and Meta-Analyses) diagram of the selection and screening process.

To ensure a comprehensive and transparent review of the literature on fNIRS in children with developmental diseases, a systematic approach was employed. The following steps outline the methodology used in this review (Figure 1).

### 2.1 Search strategy

**Figure 2:**
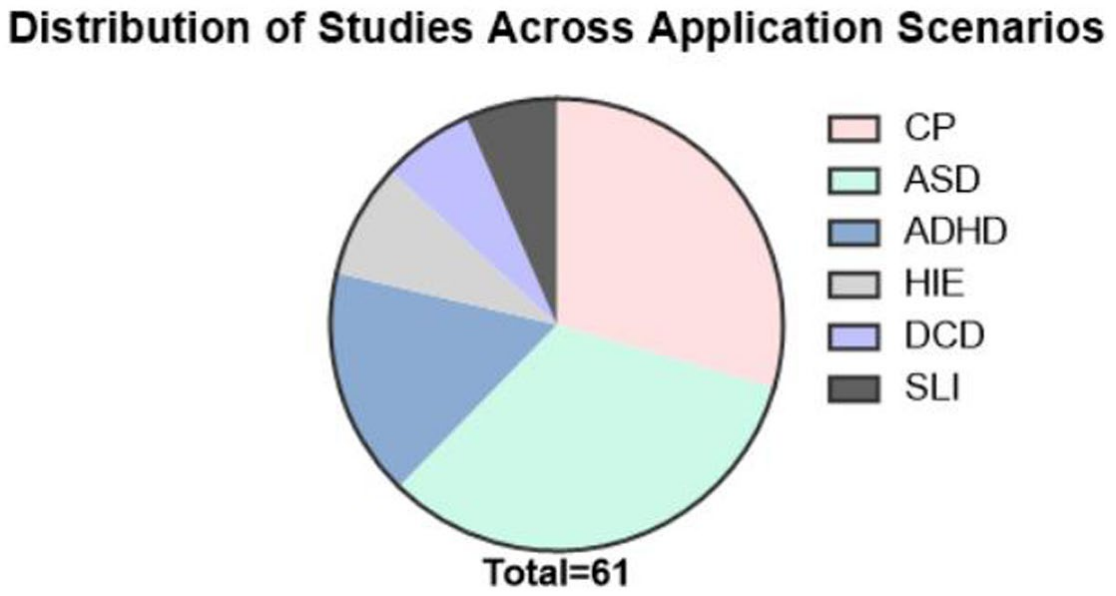
Distribution of studies across application scenarios.

The search strategy involved the use of logical combinations of the following key terms in Medline, PubMed, and Scopus to identify articles related to neuromodulation protocols combined with fNIRS: fNIRS, developmental diseases, cerebral palsy, autism spectrum disorders, neurostimulation, attention-deficit hyperactivity disorders, high-risk infants (HRIs), Developmental Coordination Disorder, Specific language impairment, transcranial magnetic stimulation (TMS), transcranial direct current stimulation (tDCS), and neurofeedback. References in the articles retrieved through manual searches were used to identify additional published articles. Studies were limited to non-conference academic publications published in English. The last date for this literature search was November 30th, 2024. Studies reporting the application of fNIRS in developmental diseases in children were included (Figure 2).

### 2.2 Inclusion and exclusion criteria

#### 2.2.1 Inclusion criteria

1. Study type: all types of research studies related to on the application of fNIRS in children with developmental diseases were considered, including randomized controlled trials, cohort studies, case–control studies, and case series.
2. Studies published in English.
3. Articles that provided detailed methodology and results related to fNIRS.
4. Studies involving human subjects, particularly children with developmental disorders.

#### 2.2.2 Exclusion criteria

1. Language: studies published in languages other than English were excluded to ensure consistency and manageability of the review.
2. Duplication: duplicate publications were removed to avoid double - counting of data.
3. Incomplete data: studies with insufficient or incomplete data to extract relevant information were excluded.

### 2.3 Data extraction processes

Two independent reviewers (Author 1 and Author 2) performed the data extraction. A standardized data extraction form was developed based on the research questions and the PRISMA - ScR checklist. The following information was extracted from each eligible study:

Study characteristics: Author(s), year of publication, study design, setting, sample size.
Patient characteristics: age, sex, primary diagnosis, severity of illness.
fNIRS: detection methods and analysis methods.
Outcome measures: specific outcome variables reported, measurement methods, and results.
Any disagreements between the two reviewers were resolved through discussion and, if necessary, consultation with a third reviewer (Author 3).

### 2.4 Framework for data synthesis

The extracted data were synthesized to identify common themes, trends, and gaps in the literature. The findings were categorized based on the type of developmental disorder and the specific applications of fNIRS. The results were analyzed to highlight the potential of fNIRS as a diagnostic and research tool in pediatric developmental disorders.

## 3 The principles and characteristics of fNIRS

The application of near-infrared (NIR) spectroscopy for detecting brain function has undergone remarkable advancements over the years. As early as 1977, Jobsis et al. (4) from Columbia University in the United States introduced the groundbreaking concept that NIR light could be used to detect changes in blood flow within relevant brain regions. Subsequently, fNIRS has garnered extensive attention and has been extensively investigated across a wide range of fields. As an optical neuroimaging technique, fNIRS measures changes in cerebral hemodynamics and oxygenation that occur in response to neuronal activity (5). The technique is founded on the principle that near-infrared (NIR) light exhibits relative transparency to human tissues and can penetrate through bones and skin. As NIR light traverses these tissues, a portion is absorbed by the medium, while the remaining unabsorbed light is detected by sensors. fNIRS capitalizes on the distinct absorption characteristics of HbO_2_ and HbR in brain tissue at varying NIR wavelengths. By measuring the intensity of diffuse light that passes through the cortex and applying the modified Beer–Lambert law, changes in hemoglobin concentration can be accurately estimated. This, in turn, offers valuable insights into brain function. Importantly, this process is intricately linked to neurovascular coupling, a physiological mechanism wherein neural activity elicits localized alterations in cerebral blood flow (6). In terms of measurement principles, fNIRS shares similarities with functional magnetic resonance imaging (fMRI) as both are functional brain imaging techniques that rely on blood flow mechanics. However, they differ in their specific approaches: fMRI detects paramagnetic changes induced by variations in hemoglobin levels, while fNIRS utilizes optical means to detect spectral absorption changes resulting from hemoglobin fluctuations (7). Therefore, fNIRS has unique technical advantages over commonly used functional brain imaging techniques. fNIRS is applicable to a wide range of people, and has better applicability to special people, such as metal implants (pacemakers, cochlear implants, etc.) in the body are not affected, and can be widely used in the clinical examination of infants and young children and patients with medical conditions; it supports long-time continuous detection and multiple examinations within a short period of time, and can be widely used in brain function monitoring and evaluation of the efficacy of the treatment; fNIRS has a good tolerance for motion interference, and has unlimited application scenarios, which makes it convenient for many patients with brain function disorders to be examined under various natural states. With the development of technology, portable fNIRS devices are smaller in size and lighter in weight, and have better tolerance to physical movement (8, 9). fNIRS is an optical device that does not interfere with electromagnetic fields, making fNIRS particularly suitable for Multi-Modality Imaging [e.g., fNIRS-fMRI (10), fNIRS-EEG (11)]. In addition, the presence of electromagnetic fields in clinical therapeutic applications does not interfere with fNIRS examinations and can be widely used in conjunction with clinical Multi-Modality nervous regulation techniques (transcranial magnetic stimulation, transcranial electrical stimulation, etc.) as a powerful tool for immediate assessment of therapeutic efficacy. In summary, fNIRS is increasingly being utilized in clinical fields such as rehabilitation, psychiatric disorders, children’s developmental disorders, and neurodegenerative diseases, thanks to its technical strengths of high spatial and temporal resolution, strong anti-interference capabilities, broad applicability to diverse populations, versatile application scenarios, and excellent mobility (12).

## 4 fNIRS applications in children with developmental disorders

### 4.1 fNIRS applications in cerebral palsy

Cerebral Palsy (CP) is a group of persistent central movement disorders and syndromes of postural abnormalities and activity limitation caused by non progressive injury to the brain of the developing foetus or infant (13). CP is the most prevalent motor disability, with an incidence rate of approximately 2–3 cases per 1,000 live births. Neurophysiological evidence indicates that children with CP often exhibit reorganization of their central motor pathways. This reorganization is typically a response to abnormal development, disease, injury, or learning processes, as the brain adapts to these challenges (14, 15). Advanced neural imaging techniques [e.g. fMRI and diffusion tensor imaging (DTI)] are commonly used for the diagnosis of pathological brain states. However, conventional brain imaging techniques typically require subjects to maintain a fixed posture and position for extended periods. This is challenging even for typically developing children, and the motor impairments associated with CP further restrict the feasibility of using these techniques to assess the anatomy and function of the brain in children with CP. The advantages of fNIRS, which is resistant to interference, simple to operate, and less affected by body posture and movement, make fNIRS more feasible for detecting children with CP. Campos et al. employed fNIRS to investigate cortical activation and functional correlates during a bilateral motor task in individuals with hemiplegia. Their findings revealed that cortical activation was significantly more pronounced in individuals with hemiplegia compared to healthy controls, particularly in both hemispheres during the bilateral asymmetric task and in the damaged hemisphere during the bilateral symmetric task. Notably, the damaged hemisphere exhibited greater activity than the non-damaged hemisphere. Additionally, higher cortical activity was correlated with more synchronous arm activation during the bilateral symmetric task. This study highlights the unique characteristics of brain activity in hemiplegic patients, such as elevated activation levels during specific functionally relevant tasks. It further suggests that fNIRS can serve as a powerful tool for assessing the degree of neurological dysfunction in affected children (16). A study evaluating the effects of progressive tests of lower limb functional muscle strength on prefrontal cortex (PFC) hemodynamics activity found lower PFC hemodynamics activation in children hemodynamicsn with CP compared with normally developing controls, suggesting that the pattern of PFC hemodynamics activity during progressive lateral step-up tests differs between children with CP and typically developing children and may be influenced by task-specific physiological and/or psychological demands (17). A study employing fNIRS to explore cortical activity changes during gait in children with spastic cerebral palsy revealed that those with spastic diplegic CP exhibited heightened activation in the sensorimotor cortex and superior parietal cortex while walking. While fNIRS may not be essential for the initial diagnosis of CP, it offers a convenient and practical approach to monitor cortical plasticity changes during and after treatment. Additionally, it plays a crucial role in assessing the severity of dysfunction and predicting outcomes in children with CP (18, 19). However, the current research on the application of fNIRS in CP is limited by small sample sizes, which precludes the assessment of potential confounding factors such as age, gender, and type of CP. Moreover, while the research samples were matched for age and gender, there is a notable absence of supplementary data collection regarding the type and timing of brain injury. Such information is crucial, as it can significantly influence functional outcomes. Additionally, cardiopulmonary indicators and emotional states, which have the potential to affect fNIRS results, have not been evaluated in the current study. Future research should address these gaps by incorporating such factors, thereby enhancing the robustness and reliability of the findings (Table 2).

**Table 2:** The representative application of fNIRS in developmental diseases.

Disease	Analysis method	Sample size	Cortical area focused	Summarizing the findings
Cerebral palsy(CP) (17)	A generalized linear regression model (GLM) was used to assess differences in PFC hemodynamic activation in the PFC across step heights	14 children with CP and 14 age- and sex-matched typically developing control children	Prefrontal cortex	Lower PFC activation in CP was maintained after statistically controlling for the number of repetitions completed at each step height.
Autism spectrum disorders(ASD) (22)	Used sample entropy (SampEn) to evaluate the characteristics of spontaneous hemodynamic fluctuations	25 children with ASD and 22 typical development children	bilateral inferior frontal gyrus and temporal cortex	The SampEn was generally lower for ASD than TD, indicating the fNIRS series from ASD was unstable, had low fluctuation, and high self-similarity. The classification between ASD and TD could reach 97.6% in accuracy.
Attention-deficit hyperactivity disorder(ADHD) (31)	task-based dynamic functional-connectivity (FC) analysis of brain signals measured by fNIRS	21 children with ADHD and 21 typical development children	prefrontal-to-inferior parietal lobes	Children with ADHD tend to show a lower probability of occurrence for the dominant connectivity state and an increased probability of occurrence for other connectivity states (states 3 and 4).
Hypoxic–ischemic encephalopathy (HIE) (44)	The RSFC patterns between newborns with HIE and healthy control subjects were compared using graph theoretical metrics.	19 neonates with HIE and 20 term-born healthy newborns	Prefrontal, temporal and parietal regions	HIE newborns showed signifcantly increased clustering coefcients, network efciency and modularity compared to controls
Developmental Coordination Disorder(DCD) (50)	Measure the activation during the performance of the Stroop task and the Wisconsin Card Sorting Task	10 children with DCD and 11 typically developing children	dorsolateral prefrontal cortex (DLPFC)	Typical developing children show DLPFC activity that changes over time in the Stroop task, with a right lateralization tendency, but do not show lateralization in the WCST. In contrast, the DCD group exhibits high and sustained activation in both hemispheres and tasks.
Specific Language Impairment(SLE) (52)	This study employed functional data analysis (FDA) methods to process fNIRS data.	15 children with SLI and 15 typically developing control children	bilateral parasylvian areas	This analysis identified significant differences between the case and control groups in the oxygenated hemodynamic mean trends in the bilateral inferior frontal and left inferior posterior parietal brain regions. We also detected significant group differences in the deoxygenated hemodynamic mean trends in the right inferior posterior parietal cortex and left temporal parietal junction

### 4.2 fNIRS applications in autism spectrum disorders

Autism Spectrum Disorder (ASD) is a group of neurodevelopmental disorders characterized by impairments in social interaction, communication, and a narrow range of interests or activities, as well as repetitive and stereotyped behavior. The etiology and pathogenesis of ASD are still unclear, and there is a lack of objective physiological indexes for the diagnosis of ASD. Currently, the diagnosis of ASD is mainly based on the diagnostic and statistical manual of mental disorders-5(DSM-5) diagnostic criteria, through behavior observation and the combination of behavior assessment scales (20). Meanwhile, the behavior manifestations of children with ASD are highly heterogeneous, and the early diagnosis of ASD is difficult, which leads to the failure of diagnosis in the early stage and the miss of optimal time for intervention in some patients (21). Several fNIRS studies on ASD have already shown that children with ASD present atypical cortical activity, even in resting state (22). Previous studies have demonstrated that as compared to typically developing (TD) children, children with ASD show weaker resting-state functional connectivity (RSFC) between the bilateral temporal lobes and asymmetry between the left and right hemispheres in the power spectrum of the low-frequency hemodynamics fluctuations (23). The other limitation of this study is that it cannot completely rule out the possibility that the differences in RSFC are due to differences in intelligence quotient (IQ). One study applied narrow-pass filters with varying bandwidths to resting-state fNIRS data and discovered that the RSFC of children with ASD was significantly weaker than that of TD children across all frequency bands. The difference in RSFC between ASD and TD children was most pronounced in the 0.01–0.02 Hz frequency band compared to other bands, suggesting that RSFC in the 0.01–0.02 Hz range may serve as a robust characteristic feature for distinguishing ASD children from TD children (24). Hirata et al. found that HbO_2_ in the left frontotemporal region of ASD patients was significantly lower than that of normal children in an emotional face recognition task by fNIRS, suggesting reduced activation of the left frontotemporal region (25). However, the study did not analyze the behavioral manifestations and brain activation of individual emotions such as sadness, anger, and fear, which is not conducive to conducting reliable statistical analysis. Future research should use a larger sample size to investigate the detailed brain activation responses to each emotional stimulus. Activation of the right inferior frontal gyrus and middle frontal gyrus in the Go/no-go task was significantly lower in children with ASD than in healthy children (26). Hyper-scanning technology, which allows for the simultaneous monitoring of brain activity in two or more people, has become a popular tool for assessing the neural characteristics of social interactions. One study used fNIRS as a technique for performing hyper-scanning to simultaneously measure prefrontal activation in 16 pairs of children with autism and their parents during a cooperative task. The study found that children with autism showed increased interpersonal neural synchronization in the prefrontal cortex during cooperative interactions with their parents, and that this neural synchronization was modulated by the children’s autism symptoms. Children with autism who had more severe symptoms showed lower levels of motor and neural synchronization when cooperating with their parents. This study provides important advances in understanding the neural correlates of social deficits in autism and provides important insights into the treatment and behavioral training of autism (27). The hemodynamics changes in different brain regions of ASD patients during social, language and executive tasks can be detected by fNIRS, which may provide a theoretical basis for further research on the pathogenesis of ASD. Meanwhile, the data of HbO_2_, HbR, total hemoglobin, and RSFC detected by fNIRS may become effective physiological indexes for the diagnosis of ASD.

### 4.3 fNIRS applications in attention-deficit hyperactivity disorders

Attention-deficit Hyperactivity Disorder (ADHD) is the most prevalent neurobehavioral disorder in childhood (27). Studies showed that the global prevalence of ADHD in children and adolescents was 8.0% (28). Nowadays, the diagnosis of ADHD is mainly based on the diagnostic criteria of DSM-V, and relies on clinical symptom observation and behavior assessment scales, which are subject to certain subjectivity, and there is still a lack of reliable objective evaluation indexes (29). The meta-analysis of fNIRS studies suggests consistent hypoactivity in the right lateral prefrontal cortex across executive function (EF) tasks associated with ADHD (30). One study measured fNIRS during a Go/No-Go task in 21 TD and 21 age-matched children with ADHD. The ADHD children tended to show decreased strong connectivity in the frontoparietal network and increased strong connectivity in bilateral medial frontoparietal cortex (31). Miao et al. (32) found that activation of prefrontal cortical functions was significantly diminished in ADHD children during the Go /no-go task and the Stroop task compared to normal children. The fNIRS was also used to detect changes in brain functional activation levels in children with ADHD before and after treatment, and Doi et al. (33) found that the performance of improved right hemisphere function after treatment was associated with activation of right prefrontal cortical function. Some children with ASD may co-morbidly suffer from inattention. fNIRS testing has also been used to differentiate ASD from ADHD. Yasumura et al. (34) found that children with ADHD had diminished functional activation of the right prefrontal cortex during the Stroop task, but this was not present in children with ASD during the Stroop task. Razoki et al. (35) used fNIRS to detect changes in cerebral blood flow in children with ADHD during biofeedback therapy to determine the effectiveness of the treatment and to develop an personalized treatment plan, and they found that the combination of fNIRS with biofeedback therapy significantly improved the therapeutic effect. As an emerging brain functional imaging technology, fNIRS plays an important role in the diagnosis, differential diagnosis, and evaluation of therapeutic effects of ADHD, and has a good application prospect. However, current research primarily focuses on the PFC, with less investigation into other cortical areas. Additionally, fNIRS is unable to detect the activity of deep subcortical structures because near-infrared light cannot reach these regions. Therefore, future studies should include a broader range of cortical areas. Moreover, this technique must be combined with other imaging methods to investigate the relationship between PFC activity and responses to stimuli.

### 4.4 fNIRS applications in high-risk infants (HRIs)

High-risk Infants (HRIs) are generally defined as (1) preterm infants; (2) infants with extremely low birth weight (ELBW) or very low birth weight (ELBW) or small for gestational age (SGA);(3) developmentally borderline children (DBCs), defined as those who are more than 2–3 months behind in their central motor functions or have abnormalities of muscle tone and reflexes or postural abnormalities;(4) neonates with special healthcare needs or dependence on life support (36). Preterm infants and hypoxic–ischemic encephalopathy(HIE) are common types of HRIs. Preterm infants are newborns born at a gestational age of less than 37 weeks. However, with the development of neonatal intensive care unit (NICU) technology and perinatal medicine, the success rate of preterm infant resuscitation has increased significantly, but surviving preterm infants face greater neurodevelopmental disorders than term infants due to the immaturity of their postnatal organs (37). Therefore, early and accurate identification of preterm infants with neurodevelopmental impairments and early and effective rehabilitation interventions using the biological basis of brain plasticity are of great importance to the prognosis of these children. Early postnatal monitoring of local oxygen saturation in brain tissue is helpful to predict the occurrence of brain injury in premature infants and determine the prognosis. If the local oxygen saturation in brain tissue is significantly reduced, it indicates that the possibility of brain injury in children is large, The local oxygen saturation level of brain tissue detected by fNIRS has high stability andrepeatability. fNIRS can be used to detect local oxygen saturation data of brain tissue in preterm infants of this period (38). At the same time, the relationship between the difference between the changes in blood oxygen levels in brain tissue detected by fNIRS and the mean arterial pressure can determine the function of cerebral blood flow autoregulation, which is important for determining the prognosis of preterm infants (39). HIE is caused by insufficient oxygen supply to the brain and disruption of blood supply during the prenatal, intrapartum or postnatal period. HIE occurs in 1 to 2 out of every 1,000 live births (40). HIE is one of the most serious birth complications affecting full-term newborns and a major cause of neonatal death and long-term disability, including cerebral palsy, epilepsy, and a variety of impairments (41). Bulgarelli et al. (42) studied the functional networks in HIE and showed that the strength of inter-and intra-hemisphere functional connectivity in the brains of HIE is reduced, especially in primary motor areas. Zhang et al. (43) reported that fNIRS examination of 13 HIE neonates showed reduced resting-state functional connectivity (RSFC) and reduced long-range connectivity compared to healthy neonates. Tang et al. (44) found utility in terms of fNIRS-detected RSFC as a potential biomarker for assessing HIE brain function. The above studies suggest that data from fNIRS assays may be a potential biomarker for early diagnosis in HRIs. fNIRS at the bedside could be complementary to other imaging modalities in terms of characterizing neonatal brain function. However, the aforementioned studies also have limitations such as small sample sizes, which to some extent limit statistical power. Moreover, current research has yet to conduct a more in-depth pathological analysis of HRIs. In future studies, fNIRS could be combined with the clinical manifestations of HRIs to assess the relationship between the lesion network and core symptoms.

### 4.5 fNIRS applications in developmental coordination disorder

Developmental Coordination Disorder (DCD) is a group of neurodevelopmental disorders that affect daily life and learning due to dysfunctional motor development (45). It is one of the most prevalent neurodevelopmental disorders in childhood, affecting 2–7% of school-age children (46). Previous studies have primarily utilized fMRI and EEG to identify neurophysiological correlates in the cerebral cortex of children with DCD. However, these techniques impose strict physical constraints that are often challenging for children with motor dysfunction to meet. As a result, detecting DCD children using these tools can be difficult. Additionally, any motor artifacts present during the examination can significantly influence the results in this population (47). fNIRS is an emerging alternative non-invasive functional brain imaging technique that can address these issues. Querne et al. (48) data on the Go-Nogo task that children with DCD exhibited more activation in the left middle prefrontal area, anterior cingulate, and inferior parietal area and less activation in the right striatum and right parietal area. Caçola et al. applied fNIRS to study the cortical activation levels of children with DCD and TD children during three tasks: Finger Tapping (FT), Curve Tracing (CT), and Paragraph Writing(PW). They found that the activation levels of different regions of the brain in children with DCD differed significantly from those of TD children when performing the above tasks, and that there were differences in the right premotor cortex and supplementary motor areas when performing FT, and differences in the right lateral dorsal prefrontal cortex when performing PW (49). Koch et al. used fNIRS to detect activation in the dorsolateral prefrontal cortex (DLPFC) in 10 children with DCD and 11 TD children performing tasks. Both groups showed similar accuracy on Stroop and Wisconsin Card Sorting (WCST), but their underlying neural activation differed. TD children modulated DLPFC activity over time and showed rightward lateralization during Stroop but no lateralization during WCST. The DCD group exhibited high and sustained activation across hemispheres and tasks, which they suggest is a compensatory effort to maintain response accuracy (50). Due to poor motor performance and participation limitations in children with DCD, the use of fNIRS can provide a window to inform neurobasic research in DCD. These results add to the body of research exploring neurological alterations in children with DCD, and establish the feasibility of using fNIRS technology with this population. The number of studies exploring the application of fNIRS in DCD remains relatively limited, and existing research often involves heterogeneous participant samples. Moreover, sudden head movements and improper placement of the head cap can introduce measurement errors. It is anticipated that technological advancements will address these challenges, thereby enhancing the reliability and accuracy of fNIRS. Ultimately, these improvements may position fNIRS as a valuable tool for both research and clinical practice in the context of DCD.

### 4.6 fNIRS applications in specific language impairment

Specific Language Impairment (SLI) is an unexplained developmental language disorder manifested by marked deficits in the acquisition and use of spoken and written language in the absence of hearing, intellectual, emotional, or acquired neurological deficits, which affects approximately 7% of the school-age population (51). Fu et al. study used fNIRS to detect differences in neural activation between children with SLI and normal children and the ability to detect differences in hemodynamic responses recorded by fNIRS by means of Functional Data Analysis (FDA), which found significant differences in mean trends in oxygenated hemodynamic in the bilateral subfrontal and left subparietal brain regions in children with SLI, and significant between-group differences in mean trends in HbR in the right posterior subparietal cortex and a significant between-group difference in mean trends in HbR in the left temporo-parietal junction (52). This study lays the groundwork for further investigation into functional brain activation in children with Specific Language Impairment (SLI) using fNIRS. It also introduces a more advanced statistical analysis method for examining the hemodynamic data collected via fNIRS. This method enables us to identify and compare the specific brain regions that are significantly engaged in grammatical comprehension ability between the two groups of participants. By employing the FDA strategy to decompose the oxygen saturation curves into mean curves and functional components to represent overall trends and variation structures, the FDA method is expected to serve as an analytical approach that can capture the overall average trends and variation trends of hemoglobin concentration over time within and between groups. Another study used fNIRS and complex network analysis to assess the dynamics of functional brain networks in dyslexia. The study found that there were differences in the topological organization and dynamics of functional brain networks, which distinguished control and dyslexic subjects, achieving an area under the ROC curve (AUC) as high as 0.89 in classification experiments (53). This indicates that fNIRS can be used to reveal differences in the neural network level of individuals with dyslexia. The article may not have detailed the potential biases in the study design, such as whether other variables that could affect brain network dynamics were considered, such as the severity of dyslexia, individual reading experience, etc. Future research needs to improve in these areas to enhance the accuracy and reliability of the study.

### 4.7 fNIRS combined with neuromodulation techniques in children with developmental diseases

Non-invasive neuromodulation techniques are biotechnological engineering methods that use non-implantable technology to physically or pharmacologically stimulate nerve fibers to regulate neuronal activity, thereby achieving a certain therapeutic effect (54). In recent years, non-invasive neuromodulation technology has developed rapidly, with technologies such as transcranial magnetic stimulation (TMS) and transcranial direct current stimulation (tDCS) being widely applied in the field of pediatric rehabilitation medicine, becoming an effective “weapon” in clinical treatment (55). However, the absence of an ability to quantify stimulation effects, particularly outside of the motor cortex, has led clinicians and researchers to pair noninvasive brain stimulation with noninvasive neuroimaging techniques. fNIRS, as an optical and wearable neuroimaging technique, is an ideal candidate for integrated use with neuromodulation techniques. A study has combined inhibitory control training with tDCS technology to intervene in the inhibitory control abilities of children with ASD, and used fNIRS to detect changes in hemodynamics in the target cortical areas during task states before and after the intervention. The research found that the training effects and transfer effects of the inhibitory control training tasks combined with real tDCS stimulation were significantly higher than those of the sham tDCS stimulation group. After receiving inhibitory control training tasks combined with real tDCS stimulation, there were significant changes in the concentration of oxygenated hemoglobin in the bilateral dorsolateral prefrontal cortex and the frontal pole area during the relevant tasks (56). This study revealed the enhancing effect of tDCS technology on the efficacy of traditional intervention methods (inhibitory control) and transfer effects (sustained attention), and emphasized the feasibility of using fNIRS neuroimaging methods to assess the effects of intervention, paving a new direction for the rehabilitation intervention methods for children with autism. Another systematic review article examined the current landscape of integrated fNIRS and TMS studies, highlighting that fNIRS, as an optical and wearable neuroimaging technology, is an ideal complement to TMS. The combination of TMS with fNIRS can assess the effects of neural stimulation in therapy. The review also discussed the application of fNIRS in monitoring changes induced by TMS and the relationship between fNIRS and cortical excitability itself (57). In summary, monitoring specific cortical response patterns related to neuromodulation with fNIRS imaging is of great significance for guiding the establishment of stimulation parameters and achieving personalized rehabilitation treatments. The application of fNIRS combined with neuromodulatory techniques such as TMS/tDCS in pediatric rehabilitation has shown potential, and these studies provide a scientific basis for further exploration of the application of fNIRS in pediatric rehabilitation. The current scope of research applications and usage, as well as the diversity in subject groups, stimulation sites, and parameters, may further exacerbate the lack of standardization between research objectives and methods. Therefore, large-scale, multicenter clinical studies with homogeneous research subjects should be conducted to clarify the role of fNIRS and neuromodulation techniques such as tDCS/rTMS in pediatric rehabilitation (Table 3).

**Table 3:** Number of studies conducting different tasks in each application scenario.

Application scenario	Cognitive tasks	Motor tasks	Social interaction	Language tasks	Executive function tasks	Neurodevelopmental monitoring tasks	Resting-state
CP	3	11	\	\	1	\	3
ASD	2	\	5	3	7	\	2
ADHD	2	1	\	\	6	\	1
HIE	\	\	\	\	\	4	1
DCD		3	\	\	1	\	\
SLE	\	\	\	4	\	\	\

The table offers a detailed breakdown of the types of tasks conducted within each application scenario. For instance, the majority of studies in Cerebral Palsy (CP) focused on motor tasks, which is consistent with the primary motor impairments associated with this condition. In contrast, studies on Autism Spectrum Disorder (ASD) and Attention Deficit Hyperactivity Disorder (ADHD) predominantly involved social/cognitive and executive function tasks, respectively. This reflects the unique cognitive and behavioral challenges faced by children with these conditions. The findings from these studies provide valuable insights into the neural correlates of these tasks and can inform targeted interventions. The chart also reveals that Specific Language Impairment (SLI) studies primarily focused on language processing tasks, highlighting the potential of fNIRS to detect neural mechanisms underlying language deficits. Additionally, studies on Hypoxic-Ischemic Encephalopathy (HIE) focused on neurodevelopmental monitoring tasks, which underscores the importance of early detection and intervention in this high-risk population.

### 4.8 The hyper-scanning based on fNIRS in children with developmental diseases

Hyper-scanning is an innovative technique that enables the simultaneous measurement of brain activity in multiple individuals during social interactions, providing valuable insights into the neural mechanisms underlying social cognition and behavior. In the context of children with developmental diseases, hyper-scanning research using advanced fNIRS has emerged as a powerful tool to explore the neural correlates of social and cognitive impairments. A study using hyperscanning technology based on functional near-infrared spectroscopy (fNIRS) has found that the inter-brain synchronization patterns of individuals with autism during social interactions are different from those of typical people, and the synchronization in brain regions such as the prefrontal cortex shows abnormalities (62). The results showed that children with ASD exhibited significantly reduced inter-brain synchronization in the prefrontal cortex compared to their typically developing peers, suggesting that the neural mechanisms underlying joint attention may be disrupted in ASD. This finding is consistent with previous research that has implicated the prefrontal cortex in social cognition and joint attention processes. In addition to ASD, hyper-scanning research has also been applied to other developmental disorders such as ADHD (58). A study by Chen et al. (59) used fNIRS hyper-scanning to examine the brain-to-brain synchrony between children with ADHD and typically developing children during a sustained attention task. The researchers found that children with ADHD showed altered patterns of inter-brain synchronization in the frontal lobe, which is involved in attention and executive function. These results suggest that hyper-scanning can provide valuable information about the neural mechanisms underlying attention deficits in children with ADHD. Moreover, hyper-scanning research can also help to identify potential biomarkers for developmental diseases. By analyzing the patterns of inter-brain synchronization, researchers may be able to identify specific neural signatures that are associated with different subtypes or severities of developmental disorders. This advancement has the potential to drive the development of more accurate diagnostic tools and personalized treatment strategies for children with developmental disorders. In the context of pediatric developmental disorders, fNIRS hyper-scanning technology has been employed to investigate interbrain neural synchronization (INS) during both cooperative and competitive tasks. For instance, Funane et al. found in their 2011 study that during a cooperative button-pressing task, there was a significant increase in interbrain neural synchronization in the prefrontal cortex areas of the interacting parties, and this synchronization was positively correlated with the quality of cooperation. Subsequent studies further explored the impact of gender differences on interbrain synchronization during cooperative tasks, discovering that opposite-sex pairs exhibited stronger interbrain synchronization during cooperation (60). Furthermore, fNIRS hyper-scanning technology has also been employed to investigate interpersonal neural synchronization during father-child problem-solving processes. Research indicates that there is a significant positive correlation between the neurobehavioral synchronization between fathers and preschool children and the scores on fatherhood role questionnaires, suggesting that a father’s attitude toward his parental role may affect the neural synchronization during father-son cooperation (61). However, it should be noted that hyper-scanning research in children with developmental diseases also faces several challenges. One limitation is the relatively low spatial resolution of fNIRS compared to other neuroimaging techniques such as fMRI, which may limit the precise localization of neural activity. Another challenge is the complexity of analyzing and interpreting the patterns of inter-brain synchronization, which requires advanced statistical methods and careful consideration of confounding factors. Despite these challenges, hyper-scanning research using fNIRS holds great promise for advancing our understanding of the neural mechanisms underlying developmental diseases in children. Future research in this domain should prioritize enhancing the spatial resolution of fNIRS, devising more sophisticated data analysis techniques, and undertaking larger-scale studies to corroborate the findings of prior investigations. Through these efforts, we can achieve a more profound understanding of the neural underpinnings of developmental disorders and ultimately develop more effective interventions to enhance the quality of life for children affected by these conditions.

## 5 Conclusion and perspective

With the advent of the Brain Initiative, an increasing number of studies are now concentrating on the neural foundations of brain science, the diagnosis and treatment of brain disorders, as well as brain-inspired artificial intelligence. fNIRS is an emerging brain imaging technology that detects brain function by non-invasive, continuous monitoring of data such as blood oxygen saturation and hemoglobin concentration in brain tissue, mainly through near infrared light. Leveraging its high safety, ease of operation, portability, low noise levels, minimal interference, high spatial and temporal resolution, broad applicability across various disease types, and minimal influence from location and posture, fNIRS has been extensively utilized in the study of childhood developmental disorders. It has emerged as a highly promising analytical and research tool, significantly advancing the continuous development of research in this field. In addition to disorders covered in the main text above, fNIRS has been widely used for epilepsy, stuttering, post neonatal surgery, visual impairment, traumatic brain injury, and child psychological research in recent years. By detecting the abnormal functional states of the brain associated with childhood developmental disorders, fNIRS has the potential to provide valuable physiological data. These data may serve as an effective biomarker for developmental dysfunctions. Their significance extends to multiple aspects of clinical practice and research, including early identification, diagnosis, assessment, treatment, efficacy evaluation, and prognosis prediction of such disorders. Furthermore, fNIRS data can facilitate a deeper exploration of the relationship between childhood developmental disorders and underlying brain mechanisms, ultimately guiding the development and refinement of clinical treatment protocols. In addition, when applying fNIRS to the study of developmental disorders in children, it is crucial to consider the applicability of the study content. Since fNIRS examines the cerebral cortex, multiple repetitions of the examination are necessary to ensure data accuracy. Moreover, the data collected by fNIRS require analysis by professionals. It is important to note that fNIRS results can be easily influenced by factors such as the subjects’ body temperature, heartbeat, and external stimuli, which may compromise data accuracy. In future studies, we should aim to expand the sample size further, minimize external interference to the subjects, and rigorously screen the original data during analysis to enhance the reliability and validity of the findings. The accuracy and precision of fNIRS in more complex clinical applications are limited by several factors. Specifically, fNIRS does not provide a comprehensive view of cortical activation, nor does it capture detailed images of deeper cortical tissues and subcortical structures. Additionally, hemodynamic responses measured by fNIRS can be influenced by systemic blood flow, further complicating data interpretation. In future research, employing precise sensor placement, anatomical co-registration, and multi-dimensional signal processing techniques can significantly enhance the sensitivity of fNIRS. These advancements have the potential to expand its utility as a versatile clinical tool for assessing brain function across a broader range of applications. As a neuroimaging tool suitable for infants, fNIRS can monitor the hemodynamic responses of the brain during the neonatal period and is a promising tool for studying the trajectory of neurodevelopment. Future research can utilize fNIRS to track the neurodevelopmental trajectories of children, providing support for early identification of neurodevelopmental disorders, elucidating the neural mechanisms behind the diseases, and predicting developmental outcomes. Further exploration of fNIRS technology’s early diagnostic and differential capabilities for children with different neurodevelopmental disorders can offer potential biomarkers for neurodevelopmental disorders. We could also initiate multicenter, large-sample clinical studies, which would help address the widespread issue of low statistical power in neuroscience research. Furthermore, the lack of standardized signal processing procedures for fNIRS data means that analysis methods vary widely across studies, thereby affecting the comparability and reproducibility of existing research. To foster future research, it is imperative to reach a consensus and standardize fNIRS signal processing methods, facilitating the replication and interpretation of results. Additionally, exploring the potential of integrating fNIRS with other imaging modalities, such as fMRI and EEG, to establish a multimodal neuroimaging approach is essential. Applying this multimodal approach to neuromodulation techniques could lead to more precise brain function detection, contributing to the development of more effective rehabilitation intervention strategies.
